# Implementing the Code of Practice on International Recruitment in Romania – exploring the current state of implementation and what Romania is doing to retain its domestic health workforce

**DOI:** 10.1186/s12960-016-0119-6

**Published:** 2016-06-30

**Authors:** Ligia Paina, Marius Ungureanu, Victor Olsavszky

**Affiliations:** Department of International Health, Health Systems Program, Johns Hopkins University Bloomberg School of Public Health, 615 N. Wolfe Street, Baltimore, MD 21205 USA; Department of Public Health and Management, Iuliu Hațieganu University of Medicine and Pharmacy, Cluj-Napoca, Romania; Cluj School of Public Health, College of Political Administrative and Communication Sciences, Babes-Bolyai University, Cluj-Napoca, Romania; World Health Organization Country Office, Bucharest, Romania

**Keywords:** Health workforce, International recruitment, Migration, Romania

## Abstract

**Background:**

The Romanian health system is struggling to retain its health workers, who are currently facing strong incentives for migration to Western European health systems. Retention issues, coupled with high levels of migration, complicate Romania’s efforts in providing basic health services for rural, underserved, and marginalized populations, as well as in achieving equitable health access for all. The WHO Global Code of Practice on International Recruitment of Health Personnel (the Code) aims to promote ethical international recruitment and health systems strengthening. We explore Romania’s implementation of the Code’s principles and recommendations.

**Methods:**

We analysed peer-reviewed and grey literature, in English and Romanian, and sought secondary data from the websites of Romania’s largest medical universities. The analysis was guided by the following themes and recommendations in the Code: health personnel development and health systems sustainability, international cooperation, data gathering, information exchange, and implementation and monitoring of the Code.

**Results:**

Romania’s implementation of the Code was observed to be limited. Gaps were identified with regards to several aspects of the Romanian health system, including the lack of support to health personnel training, recruitment, and retention in order to increase the appeal for health providers to practice in Romania and in underserved areas. In terms of international cooperation, the Code recommends various policy instruments to guide recruitment, including bilateral agreements. However, we could not determine which of these instruments were used as a result of the Code and whether or not they were effective. We identified little evidence of initiatives for health workers’ professional and personal support. Insufficient data and few information exchange platforms exist on health workforce issues, hindering active sharing of data on migration with European Union and WHO audiences. We could not identify any evidence of monitoring of the Code’s implementation to date.

**Conclusions:**

In the absence of major system reforms, health workers will continue to migrate to urban areas and abroad. Romanian policymakers should address more of the Code’s recommendations by developing a national policy for human resources for health, a central database to aid health workforce planning and management, stronger platforms for information exchange and civil society engagement, and updated and transparent bilateral agreements.

## Background

Worldwide, many countries are experiencing health workforce imbalances that are leading to inequities in terms of access and availability of care. The World Health Organization (WHO) estimates that, in 2006, there was a global shortage of 4.2 million health workers [1]. While sub-Saharan Africa suffers the most critical shortages, Europe is not immune, as the patterns of the global health workforce market are intricately interdependent. At the European Union (EU) level, a shortage of 1 million health workers is expected by 2020 [2].

In response to increasing health workforce migration and its contributions to the shortage of health personnel in source countries, in 2010, WHO elaborated the Global Code of Practice on the International Recruitment of Health Personnel (the Code) [3]. The Code, “*establish*[es] *and promote*[s] *voluntary principles and practices for the ethical international recruitment of health personnel and facilitate*[s] *the strengthening of health systems,* […] *serve*[s] *as a reference for Member States in establishing or improving the legal and institutional framework required for the international recruitment of health personnel,* […] *provide*[s] *guidance that may be used* […] *in the formulation and implementation of bilateral agreements and other international legal instruments,* [… and] *facilitate*[s] *and promote*[s] *international discussion and advance cooperation on matters related to the ethical international recruitment of health personnel as part of strengthening health systems, with a particular focus on the situation of developing countries*” [3, 4]. The Code, which was adopted by all 193 WHO Member States, seeks to redress imbalances among health workers by raising issues of human rights, according to health, equity, and social justice [3]. The Code is voluntary, but “*Member States and other stakeholders* [such as non-governmental organizations] *are strongly encouraged to use the Code*” [3].

In 2014, WHO issued the first report on the implementation of the Code, based on the information submitted by its Member States through the related Code National Reporting Instruments [5–7]. Out of the 56 responses received, 40 originated in European countries. Thirty-seven Member States reported having taken actions to communicate and share information across sectors on health worker recruitment and migration issues, and only nine Member States reported good practices that have been encouraged and promoted among recruitment agencies [7]. Overall, the report highlighted that Member States had not systematically applied the Code and that significant gaps still remained in ethical international recruitment and local systems strengthening [7].

Romania, an upper middle-income country and member of the EU since 2007, deals with a health workforce crisis of its own linked to migration. It is currently a key source country for Western Europe, with many Romanian doctors, nurses, and other health professionals migrating to work in the United Kingdom, France, Germany, Italy, and Spain, among others. Despite the ‘brain drain’ having been a common trend since the fall of communism in 1989, the phenomenon seems to have grown after EU accession in 2007 [8]. Indeed, the Romanian College of Physicians reported that “*between 2007 and 2013, 14,000 medical doctors left their jobs in the national public health system and choose to practice abroad*” [7]. This raises concerns since, in 2012, less than 40,000 medical doctors were licensed to practice in Romania [7]. Romanian health workers’ mobility is affected by both push and pull factors; the push factors generally relate to challenges and insufficiencies within the Romanian health care system, whereas the pull factors relate to the prosperity of Western European health systems, which translate into much higher salary figures than those currently feasible in Romania. Within Romania, internal migration from rural to urban areas persists. Therefore, poor and remote populations are disproportionately experiencing the combined effects of migration patterns and the local shortcomings in health workforce management [9]. Rich segments of the population can seek care in the growing private sector, pay informal charges, or even seek care abroad. However, rural populations, which make up almost half of the total population [10], as well as marginalized urban populations (e.g. the Roma), are left without many options [9].

Romania has approximately 2.5 practicing doctors and 5.8 practicing nurses per 1000 inhabitants; nevertheless, these numbers are low compared to other European countries [11]. Recently, the Romanian College of Physicians warned that Romania had reached critically low numbers of practicing doctors [7]. Moreover, some rural and remote villages, where poverty rates can be twice as high as in urban areas, do not have a full-time medical assistant/nurse or a doctor [12]; for example, in 2005, a family doctor was not available in 98 localities [13]. Further, Romania has an overall surplus of general practitioners, 63 % of whom practice exclusively in urban areas, and yet there is a dearth in cardiology, intensive care, and surgery specialists, with only 20 % of vacancies being filled [13]. Thus, one-third of Romanians do not have access to specialists to treat the increasing burden of non-communicable diseases (e.g. cardiovascular disease, diabetes, and emergency medicine and intensive therapy care) [13]. Health promotion is another important area lacking attention in rural populations. For example, in rural Transylvania, one in two people are not reached by health promotion campaigns [14]. The lack of access to adequate health care, as well as the overall aging trends across Europe, are having detrimental effects on the health of the Romanian population and have led to Romania having some of the worst health and health system statistics in Europe [10].

Despite the current crisis, there is little documentation on whether and how Romania has been following the principles and recommendations outlined in the Code in order to manage migration and to improve health worker retention. Currently, Romania does not have a “*valid and reliable monitoring system on health professional mobility*” [7, 15]. Furthermore, Romania did not submit a National Reporting Instrument as part of the first round of the Code’s monitoring [7]. Herein, we explore whether and how Romania has adhered to the Code by documenting the policies and measures implemented to strengthen the health workforce and the health system, to incentivize health workers to remain and practice in Romania, and to gather and exchange information at national and international levels. We also reflect on how relevant and effective the Code has been in Romania to date and propose recommendations for advancing efforts to address the health worker crisis in Romania.

## Methods

The WHO Global Code of Practice on International Recruitment of Health Personnel proposes a set of voluntary principles and practices to help Member States ethically recruit health professionals and strengthen health systems [3]. These focus on ethical international recruitment, health workforce development and health systems sustainability, fair treatment of migrant health personnel, international cooperation, support to developing countries, data gathering, and information exchange. We focused on a subset of these, including health workforce development and health systems sustainability, international cooperation, data gathering, and information exchange. These areas served as a framework for guiding our analysis and organizing literature review findings.

We conducted a search of English and Romanian-language literature on migration of Romanian health professionals and health workforce management in Romania. Peer-reviewed literature was extracted from several databases, including PubMed, EMBASE, DOAJ, CIAO, and JSTOR. In addition, grey literature was also reviewed from the following sources: Organization for European Co-operation and Development, International Organization for Migration, European Observatory on Health Systems and Policies, EU, and WHO. Policies and reports were also extracted from the website of the Romanian Ministry of Health. Secondary data, especially for the education component, was sought on Medical University websites, focusing on Romania’s major medical schools (Bucharest [16], Timişoara [17], Cluj [18], Iaşi [19], and Târgu Mureş [20]). Data was also examined to assess how the admission of students from rural areas is documented and whether medical curricula include rural health components. To the best of the authors’ knowledge, Romania does not directly track international migration and professional associations’ registries, such as that of the College of Physicians, do not track migrating professionals [21]; therefore, it was not possible to develop a snapshot of migration or to examine trends. Since 2007, international migration can be indirectly estimated through tracking of the number of Certificates for Recognition of Professional Qualifications issued by the Ministry of Health [22]. For example, in 2010, the Ministry issued more than 300 certificates per month [15], though another source estimates the number to be lower, at approximately 200 per month [7]. However, this data unreliably estimates migrating health providers – those who request this certificate might have migrated prior to requesting it or might never do so. Romania has not, to date, conducted a human resources for health audit.

## Results and discussion

Information and data with regards to strategies for mitigating migration and promoting rural retention in Romania were difficult to find. Few related peer-reviewed publications were identified on this topic of interest, and, therefore, the present findings rely mostly on the grey literature, including reports and case studies in which Romania was featured. Below, we synthesize and document the policies and measures currently identified in Romania, as linked to the Code principles and practices described above.

### Health workforce development and health systems sustainability

Article 5 of the Code encourages Member States to “*consider adopting and implementing effective measures aimed at strengthening health systems, continuous monitoring of the health labour market, and coordination among all stakeholders in order to develop and retain a sustainable health workforce responsive to their population’s health needs*” [3, 23]. Through this recommendation, the Code draws attention to Member States tackling the underlying push and pull factors of migration. Furthermore, in Article 5.4, the Code states that “*an appropriate health workforce, should be educated, retained, and sustained for the specific conditions of each country, including areas of greatest need and that all Member States should strive to meet their health personnel requirements with their own human resources for health*” [3, 23]. The Code then refers to the WHO Global Policy Recommendations on Increasing Access to Health Workers in Remote and Rural Areas through Improved Retention, which specifies key recommended interventions [24]. We grouped our findings according to these interventions.

#### Education

Romania has a well-established medical training system, primarily comprised of 12 public medical schools and only one private medical university [25]. All of these universities are located in major urban areas and no training programs have been identified in rural areas. In our examination of the medical curricula from the top five medical universities in Romania, located in Bucharest [16], Timişoara [17], Cluj [18], Iaşi [19], and Târgu Mureş [20], we could not find evidence of an explicit focus on rural issues. Specifically, we did not identify any evidence of rural clinical rotations during medical training, whether mandatory or not, nor did we find courses focusing on rural health; it is possible that the topic might be covered to some extent as part of the public health class, however, this could not be determined based on the available information. Documenting the recruitment and admission of students from rural areas or underrepresented populations was also examined. Since 2012, the Romanian Ministry of Education, Research, Youth, and Sport mandates reporting of the number of admission slots allocated to Roma candidates. However, during the academic year 2012/2013, the Medical University Victor Babeş in Timişoara, for example, only had three registered Roma students out of a class of 580, and the Medical University in Târgu Mureş had two registered Roma in a class of 440 students [26]. Medical school admission records did not track students from rural areas. Tracking students from rural, poor, and underserved areas and populations is an important first step. However, given the low numbers declared, greater measures should be taken at all levels to provide support both to the Roma students applying and to the Medical Universities training them.

In addition to pursuing medical education within the national system, Romanian students can also seek to study abroad. Since 2005, Romanians can pursue their studies in Western Europe or North America, with many of these students seeking medical training. However, the scholarships received for foreign study are given only on the condition that students return to Romania after graduation and work in public service management positions for a minimum of 3–5 years [27]. This program does not currently stipulate requirements for those returning from medical training abroad to complete their mandated service in underserved or rural areas.

#### Regulation

National, regional, and global reforms should all be considered in the context of Romania’s efforts to recruit and retain health workers to rural and underserved areas. Distinct regional and global reforms could not be identified, but related international cooperation mechanisms are discussed ahead. We identified only one national level initiative – the Romanian Ministry of Health launched an initiative for residency reform in 2009 [28]. According to this initiative, medical graduates can select one of two distinct residency tracks – one which allows residents to bid on a particular residency location (the location track – most competitive) and one which allows residents to qualify with lower scores, with no control over the residency location (the position track – less competitive). Residents bidding through the position track are usually placed in a rural or underserved area and are bound by contract to remain in this position for a duration equivalent to that spent in medical training [28]. The residency initiative has not been formally evaluated, but anecdotal evidence points to loopholes that could undermine the initiative’s objective to post residents in underserved areas. For example, residents could negotiate with local hospital officials to get their way out of their compulsory contract before the period is completed, or, conceivably, even before they are sent to their post. The residency reform initiative was initiated before the Code’s implementation began and provides an example of a regulatory mechanisms that can be adapted and evaluated to support the Code’s principles and ensure effectiveness.

#### Financial incentives

Medical professionals in Romania have few financial incentives to work in the Romanian health system, particularly in light of the potential pay and incentives available in Western European countries. Health worker wages have been historically low in Romania, compared to those in other EU countries. For example, a resident in Romania earns around €200, whereas residents in other EU countries can earn, on average, around €1100. Similarly, specialists that would earn around €495 in Romania, could earn, on average, almost €8000 in other EU countries [9, 27]. Nurses’ salaries are equally low, at less than €300 per month. These staunch differences arise from broader health system financing issues. Romania has a historically low total health expenditure as a percentage of the GDP. Even recently, in 2012, the total health expenditure in Romania WHO estimates accounted for 5.1 % of the GDP, a decrease from 2011 (5.6 %) and 2010 (5.9 %) [29]. At the same time, neighbouring countries such as Hungary, Poland, and France were allocating 8, 7, and 11 % of their GDP to health, respectively [29]. Public providers are believed to often also practice in the private-for-profit sector, either in their own clinics or at one of the many new private hospitals. However, private practices are commonly located in urban areas, where it is easier to find these opportunities and profits can be higher. In addition, the austerity measures initiated in response to the 2008 economic crisis continue to reverberate in the health system, including a health sector hiring freeze and civil service reforms affecting all public sector medical personnel and resulting in a 25 % salary cut, which is slowly being re-adjusted [7].

Since 2008, family physicians are incentivized to practice in rural areas through the Framework Contract, the mechanism through which they are contracted by the National Health Insurance Fund [30]. Family practitioners are reimbursed on a point system, using a formula that sums up points received for each patient in one’s practice (per capita) and those received for a particular service (per service). The total number of points is then multiplied by a monetary value, which is set by the National Health Insurance Fund on a yearly basis. Depending on their practice location and their work environment, family practitioners are entitled to inflated points – and therefore higher pay. The five domains towards which doctors can earn points include their practice’s location (e.g. distance to closest urban setting); the conditions under which medical care is provided (e.g. potential for high patient load, based on population density); service delivery and referral support (e.g. distance between location of practice and the closest emergency unit); population socioeconomic level (e.g. proportion of patients who receive subsidized health insurance); and the number of insured patients [30]. If a provider gains between 51 and 57 points, the monetary value of those points is increased by 82–100 %. The fewer the points, the smaller the multiplier. Primary care offices located in the Danube Delta, which is classified as a hardship area, benefit from a 100 % increase, essentially doubling the monetary value of providers’ points. Therefore, the income that family practitioners could earn there (i.e. in a rural, remote, and poor area) is comparable to that of their peers working in wealthier, better connected urban areas [30]. Nevertheless, a recently published study on the recruitment and retention of health personnel established that, to date, these incentives have not led to an increase in the number of family physicians or general practitioners in rural and underserved areas [31]. Finally, we could not identify similar incentives for other types of health workers or specialists.

#### Professional and personal support

Professional and personal support activities refer to non-financial incentives for rural recruitment and retention. In terms of professional support, health workers with leadership and management roles, such as health facility managers, have little control over managing the workforce and seldom the flexibility to implement performance-based management. Furthermore, planning and evaluation can be mistrusted, due to its reputation as ‘communist’ [32]. Health workers are often mistrusted personally due to Romania’s history of high corruption and informal payments in the health sector. Given these patient and society perspectives, health workers generally enjoy less prestige and a lower social position than in the past [33].

The lack of support also manifests itself through the absence of stimulating career development activities. For example, young medical school graduates can pursue career advancement by completing residency and advanced specialist training. These can be technically completed only within 10 years following medical school graduation, leaving doctors with few further career development options (with the exception of academic titles and administrative positions within their facilities) until they retire (Authors’ own observations). Due to the aforementioned examples, the lack of professional support at the individual and system levels has become an overall push factor for medical professionals, and likely for other types of health workers as well.

### International cooperation

We identified several approaches to international cooperation that could be related to the Code’s implementation: bilateral agreements, EU-wide policies, and research and advocacy efforts engaging civil society.

The Code recommends bilateral agreements as policy instruments for managing the recruitment of health professionals and facilitating synergies between the signatory countries [3]. At the regional level, since 1990, Romania has signed 11 bilateral agreements with countries for which it serves as a source country for health professionals [27, 34]. For example, Romania signed bilateral agreements with Germany, Greece, Spain, and Italy in order to allow Romanian nursing cadres to practice there [27]. Some of these agreements were signed between sub-national entities (e.g. between Italian and Romanian provinces [34]), while others were signed at the national level (e.g. between Germany and Romania in 2005, to manage the recruitment of foreign nursing aids [27, 34]). Romania and the Republic of Moldova, which serves as a source country for Romania, signed a bilateral agreement in 1998; this agreement was amended by a protocol issued in 2010 and is currently due for renewal [35]. We could not determine which of these agreements were initiated by Romania and which by other countries, or whether any of these bilateral agreements have any monitoring and evaluation components. Furthermore, none of these could be identified in their original form, nor directly linked with the Code’s implementation.

More broadly, especially after acceding to the EU, Romania has been engaging in discussions related to standardizing competencies and medical training education across the EU to facilitate “*free movement of labor*” [36]. Under this cornerstone EU principle, EU citizens can seek employment in another EU country without the need of a work permit and can reside there, as well as “*enjoy equal treatment with nationals in access to employment, working conditions, and all other social and tax advantages*” [36]. The recognition of qualifications and training consequently becomes an important policy harmonization activity. For health professionals, this has been facilitated by European regulations that stipulate the automatic recognition of qualifications (e.g. Directive 2005/36/EC33), though the recognition is not ‘automatic’ everywhere and the related administrative burden varies by country [27]. Furthermore, while medical diplomas are recognized in France, for example, they are not considered to be equivalent to those obtained through French medical education [27]. As of 2014, this Directive was under review [27] and, therefore, further linkages to the Code’s implementation remain to be seen.

Finally, there are several EU-wide research and advocacy efforts in which Romania participates. For example, the Health Workers for All is an initiative funded by the EU/Europe Aid, aimed at enhancing the collaboration and exchange of good practices on health workforce management among the participating countries [37]. Romania is a partner in this project, represented by the Center for Health Policies and Services in Bucharest. The initiative has been fruitful, having contributed to raising awareness about the Code and health workforce challenges, as well as identifying good practices for health workers’ mobility. Romanian researchers are also part of the EU Joint Action on Health Workforce Planning and Forecasting [38]. The Joint Action, co-funded by the European Commission, aims at creating a platform for collaboration among the member states in terms of planning and forecasting methodologies.

### Data gathering and information exchange

Data gathering and information exchange are two cornerstone principles of the Code, premised on the idea that a solid evidence base is needed to develop effective policies to address health workforce migration, as well as developing sustainable health systems. For example, Article 3 of the Code emphasizes the need to have “*effective gathering of national and international data, research, and sharing of information on international recruitment of health personnel*”, as well as the importance of policies based on sound evidence; having strong health personnel information systems – including health personnel migration and its impact on health systems; collecting, analysing, and translating data into effective health workforce policies and planning; and strengthening research personnel [3, 23].

With regards to data gathering, little data is available on Romanian health workforce migration and on the health workforce in general. The main indicator is the ‘intention to leave’ collected by the Romanian College of Physicians [15]. In 2007, for example, 10.2 % of practicing medical doctors applied for diploma verification (compulsory in order to receive acceptance to leave the system) [15]. Further, as mentioned above, tracking of medical student and physician characteristics is scarce, as they pertain to recruitment and retention in rural and underserved areas. Additionally, despite a large number of medical professionals having already migrated, it is unclear whether any of them returned, from where, and where they are currently employed (i.e. health or non-health; public or private sector). A human resource information system or audit has never been organized for Romania, and therefore, even less is known about non-physician medical professionals.

Article 7 of the Code encourages Member States to promote national and international exchange of both qualitative and quantitative information on the health workforce, as well as on the implementation of the Code [3, 23]. In addition, Member States are encouraged to share at least a minimum data set with WHO, based on WHO-issued guidelines of key variables and definitions [39]. According to these guidelines, countries are to trace information about generalist and specialist medical doctors, nurses, nursing professionals, midwives, midwife professionals, and associated professionals [39]. The information to be traced includes a range of indicators, such as the countries of first and last qualification, current employment status, working hours, and duration of stay [40]. A national authority is designated to facilitate international communication through periodic reports such as the National Reporting Instrument [3, 6, 23]. In Romania, the designated national authority is the Human Resources Unit of the Ministry of Health, the same body that is in charge of organizing the medical residency process and releasing conformity certificates or a diploma verification for health professionals that intend to leave Romania [22]. However, as mentioned in the introduction, Romania has not yet submitted the National Reporting Instrument.

There are further avenues through which Romania has shared information about its health workforce on the international arena. Data concerning Romania does appear in a number of recent European publications – Romania is one of the 17 countries featured in a recent publication on the mobility of health professionals [9], it is one of six countries to be featured in the first review of the Code’s implementation [7], and is one of the countries featured in recent reports by the Health Workers for All advocacy initiative [5, 37]. The latter also features a brief report of ‘good practice’ in ethical international recruitment between Romania and Bulgaria. The Călăraşi County Emergency Hospital recruited Bulgarian, to work contract shifts in Romania in addition to their original full-time positions in Bulgaria. This program allowed Bulgarian doctors to augment their income by working in the region (rather than Western Europe), helped to compensate for the shortage in Romanian doctors, and facilitated learning exchanges [40, 41].

### Limitations and future work

Our study was limited by the low volume of publicly available data on the topics explored; few peer-reviewed publications were identified and grey literature reports where Romania was profiled varied in level of detail. Furthermore, online searches were limited by the amount of information that is publicly disclosed on any institution’s website. For example, medical school websites might not be updated to reflect the information reviewed herein, or, alternatively, might not publicly disclose certain information. Due to funding and time constraints, we could not make specific, formal data requests. However, these would be necessary in future studies. Future research should focus on highlighting good practices when implementing the Code [40, 41]. Local adaptation and coping mechanisms could provide insights into how to best adapt the policy framework to remove some of the key bottlenecks faced by facilities on the ground. Furthermore, more studies are needed to understand doctors and nurses that work abroad temporarily, as well as health workers’ preferences for remaining in Romania and working, temporarily or permanently, in rural and underserved areas. Since the private health sector plays an increasingly prominent role, more research is required on the incentives it offers to health providers, such as dual practice opportunities, better working conditions, and prestige. It is critical that bilateral agreements signed to date are evaluated and that recruitment agencies and their strategies are documented and linked to policy actions, both in Romania and the EU. Finally, according to the authors’ knowledge, no evidence was available on the implementation of the principles of the Code not discussed herein.

## Conclusions

In an increasingly interconnected world, health workers will continue to migrate. Only if health systems create favourable conditions for their health workers, will doctors, nurses, and other health professionals feel incentivized to practice in a particular country or geographical area. Furthermore, a strong health workforce policy and management is required to mitigate the negative consequences of migration and other phenomena, which can affect equity and universal access to care.

Very little data on health workforce migration exists in Romania, hindering the assessment of what the country is currently undertaking to implement the Code. Based on the information gathered, Romania could increase its efforts to adhere to the principles and recommendations made in the Code and to strengthen its health workforce overall. The development and implementation of a health workforce policy would be a first step towards achieving a sustainable health system and health workforce, setting a strategic vision for health workforce planning and management, and providing a framework to address current shortfalls. The Ministry of Health recently launched its new Health Strategy 2014–2020, with human resources policy and management being among the targeted objectives (Strategic Objective 5.2 – The implementation of a sustainable human resources for health policy) [42]. During the development and implementation of this policy, the WHO Country Office Romania, as well as other international or national partners, could play an active role in supporting the integration of the principles included in the Code.

Romania should further strengthen the availability of health workforce data, which should be readily disaggregated by geography (i.e. rural/urban) and cover migration issues. This would facilitate policy and decision-making within Romania, as well as the assessment of Romania’s adherence to the Code and its implementation. Furthermore, a centralized database for health workforce data that tracks health worker mobility, as well as more research on health workforce issues, would develop a sounder evidence base for decision-making. The Ministry of Health, along with professional associations, should lead the development of such a database as well as data sharing coordination with relevant institutions from the major recipient countries of Romanian health providers. These efforts and linkages could help paint a holistic picture of health workforce migration. While civil society has been engaged on the topic at the European level, for example, through the Health Workers for All initiative [37], little appears to have been initiated at the country level. Greater civil society engagement in the health system would help foster accountability, create a platform for information exchange, and allow a platform for multiple voices to be heard in decision-making.

At the European level, there should be a greater recognition of the domino effect in international migration – while Romania has signed several bilateral agreements as a source country, it has not initiated any as a destination country – despite its increasing role in the latter. Furthermore, the monitoring and evaluation of bilateral agreements should be given more emphasis in order to understand whether and how they work as policy instruments.

As suggested by our analysis, Romania is currently not doing enough to recognize and address the challenges of its increasingly mobile health workforce. However, we propose that the Government of Romania should follow the Code in order to better understand and document the dynamics of its health workforce, to forecast its needs, and to implement health worker retention and motivation strategies.
